# Supplementary material for: Climate change favours connectivity between virus-bearing pest and rice cultivations in sub-Saharan Africa, depressing local economies
Source: PeerJ. 2021 Nov 19;9:e12387. doi: 10.7717/peerj.12387 (PMC8607929; doi:10.7717/peerj.12387)
Supplement: Supplemental Information 1 [file peerj-09-12387-s001.docx]

**Table S.1** – Occurrences of *Chaetocnema pulla* group by Biondi & D’Alessandro (2008) and personal data (M. Biondi).

| **Species** | **Latitude** | **Longitude** |
| --- | --- | --- |

| *Chaetocnema nkolentangana* | 4.18 | 29.53 |
| --- | --- | --- |
| *Chaetocnema pulla* | 12.39 | -16.19 |
| *Chaetocnema pulla* | 12.27 | -14.45 |
| *Chaetocnema pulla* | 12.52 | -14.13 |
| *Chaetocnema pulla* | 8.43 | -13.22 |
| *Chaetocnema pulla* | 8.33 | -13.18 |
| *Chaetocnema pulla* | 8.27 | -13.07 |
| *Chaetocnema pulla* | 8.22 | -12.67 |
| *Chaetocnema pulla* | 9.48 | -12.23 |
| *Chaetocnema pulla* | 8.88 | -12.05 |
| *Chaetocnema pulla* | 9.13 | -11.97 |
| *Chaetocnema pulla* | 9.63 | -11.87 |
| *Chaetocnema pulla* | 9.58 | -11.58 |
| *Chaetocnema pulla* | 7.62 | -11.33 |
| *Chaetocnema pulla* | 9.33 | -11.33 |
| *Chaetocnema pulla* | 7.69 | -5.03 |
| *Chaetocnema pulla* | 4.22 | 8.98 |
| *Chaetocnema pulla* | 4.22 | 9.13 |
| *Chaetocnema pulla* | 4.45 | 9.25 |
| *Chaetocnema pulla* | 4.95 | 9.93 |
| *Chaetocnema pulla* | 2.07 | 11.64 |
| *Chaetocnema pulla* | -33.97 | 18.42 |
| *Chaetocnema pulla* | 3.65 | 18.63 |
| *Chaetocnema pulla* | -18.17 | 23.43 |
| *Chaetocnema pulla* | -17.48 | 24.28 |
| *Chaetocnema pulla* | 0.22 | 24.45 |
| *Chaetocnema pulla* | 2.81 | 24.75 |
| *Chaetocnema pulla* | 1.37 | 27.58 |
| *Chaetocnema pulla* | -24.35 | 27.63 |
| *Chaetocnema pulla* | -1.86 | 28.45 |
| *Chaetocnema pulla* | -2.23 | 28.78 |
| *Chaetocnema pulla* | -32.28 | 28.85 |
| *Chaetocnema pulla* | 0.88 | 28.87 |
| *Chaetocnema pulla* | -2.55 | 28.98 |
| *Chaetocnema pulla* | -3.35 | 29.15 |
| *Chaetocnema pulla* | 0.53 | 29.15 |
| *Chaetocnema pulla* | -3.37 | 29.35 |
| *Chaetocnema pulla* | 0.40 | 29.6 |
| *Chaetocnema pulla* | -30.53 | 29.68 |
| *Chaetocnema pulla* | -30.57 | 29.72 |
| *Chaetocnema pulla* | 0.44 | 29.82 |
| *Chaetocnema pulla* | 1.12 | 30.27 |
| *Chaetocnema pulla* | -1.58 | 30.6 |
| *Chaetocnema pulla* | -22.45 | 31.17 |
| *Chaetocnema pulla* | -25.07 | 31.6 |
| *Chaetocnema pulla* | 0.02 | 32.32 |
| *Chaetocnema pulla* | 14.42 | 33.51 |
| *Chaetocnema pulla* | 8.24 | 34.58 |
| *Chaetocnema pulla* | 7.89 | 36.84 |
| *Chaetocnema pulla* | 1.22 | 36.90 |
| *Chaetocnema pulla* | 10.00 | 37.33 |
| *Chaetocnema pulla* | 11.56 | 37.37 |
| *Chaetocnema pulla* | 12.6 | 37.47 |
| *Chaetocnema pulla* | 9.10 | 37.86 |
| *Chaetocnema pulla* | 7.63 | 38.68 |
| *Chaetocnema pulla* | -7.98 | 38.78 |
| *Chaetocnema pulla* | 14.18 | 38.88 |
| *Chaetocnema pulla* | 13.50 | 39.47 |
| *Chaetocnema pulla* | 11.23 | 39.57 |
| *Chaetocnema pulla* | -2.17 | 41.00 |
| *Chaetocnema pulla* | -12.80 | 45.15 |
| *Chaetocnema pulla* | -14.20 | 48.08 |
| *Chaetocnema pulla* | -13.33 | 48.27 |
| *Chaetocnema pulla* | -17.48 | 48.45 |
| *Chaetocnema pulla* | -17.72 | 48.65 |
| *Chaetocnema pulla* | -17.37 | 49.40 |
| *Chaetocnema pulla* | -16.92 | 49.57 |
| *Chaetocnema pulla* | -15.43 | 49.73 |
| *Chaetocnema pulla* | -16.28 | 49.82 |
| *Chaetocnema pulla* | -16.33 | 49.83 |
| *Chaetocnema pulla* | 14.00 | 38.20 |
| *Chaetocnema pulla* | 11.50 | 37.40 |
| *Chaetocnema pulla* | 11.60 | 39.20 |
| *Chaetocnema pulla* | 11.20 | 39.40 |
| *Chaetocnema pulla* | 10.40 | 37.20 |
| *Chaetocnema pulla* | 9.30 | 38.50 |
| *Chaetocnema pulla* | 8.53 | 30.10 |
| *Chaetocnema pulla* | 8.24 | 34.60 |
| *Chaetocnema pulla* | 13.28 | -14.21 |
| *Chaetocnema pulla* | 12.40 | -15.81 |
| *Chaetocnema pulla* | 9.22 | -11.22 |
| *Chaetocnema pulla* | 8.47 | -13.40 |
| *Chaetocnema pulla* | 8.37 | -13.17 |
| *Chaetocnema pulla* | 4.02 | 9.19 |
| *Chaetocnema pulla* | -4.88 | 38.28 |
| *Chaetocnema pulla* | -31.63 | 29.54 |
| *Chaetocnema pulla* | -33.97 | 18.42 |
| *Chaetocnema vanschuytbroecki* | 0.31 | 29.75 |


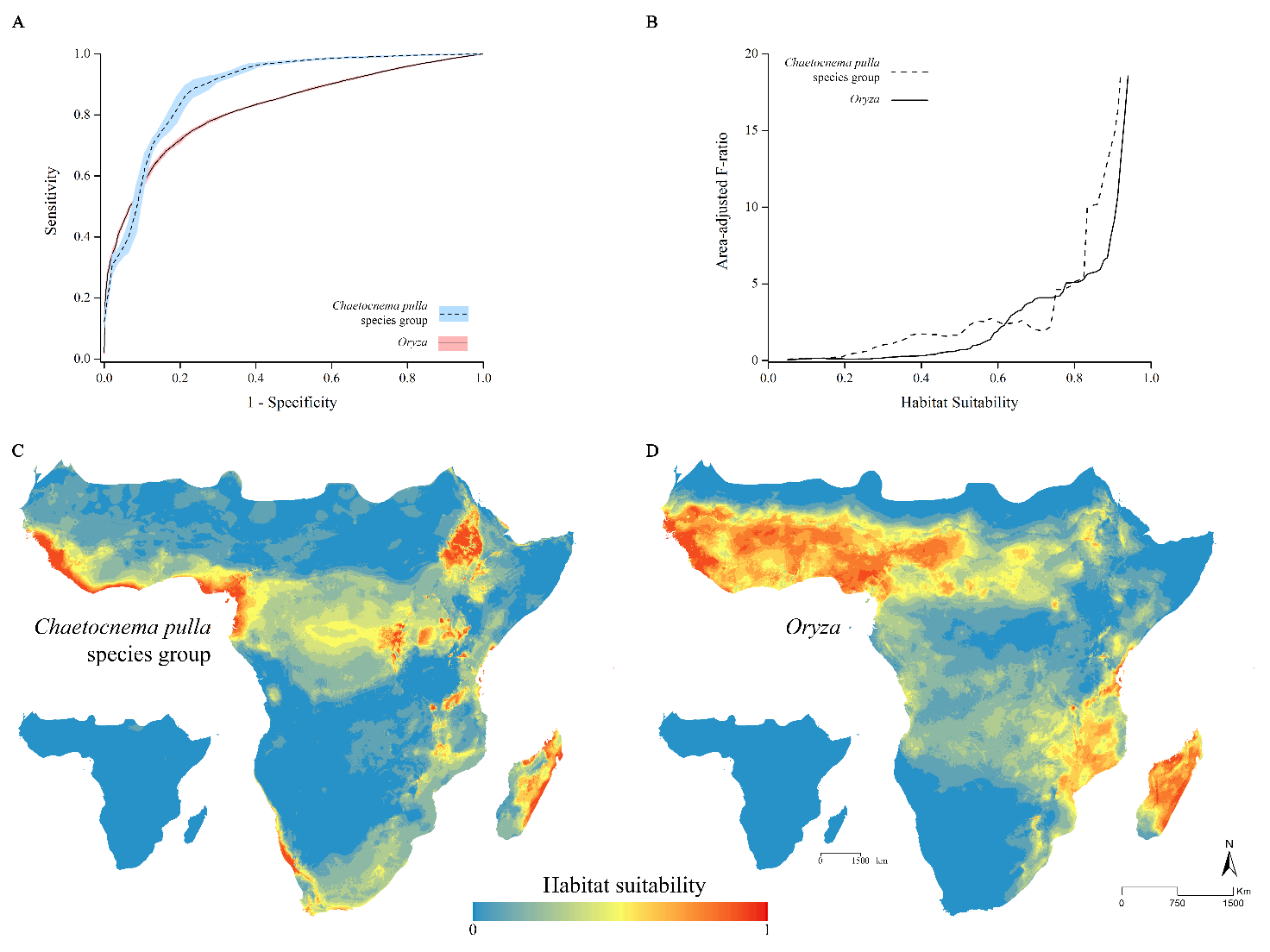


**Fig. S.1 - Models' discrimination performance and main outputs.** The Receiver Operating Characteristic (ROC) curve (A) and the continuous Boyce index curve (B) obtained from the models' calibration performed over current climatic conditions for *Chaetocnema pulla* species group (C) and *Oryza* sp. (D) (larger maps: habitat suitability; smaller maps: the corresponding standard deviation).


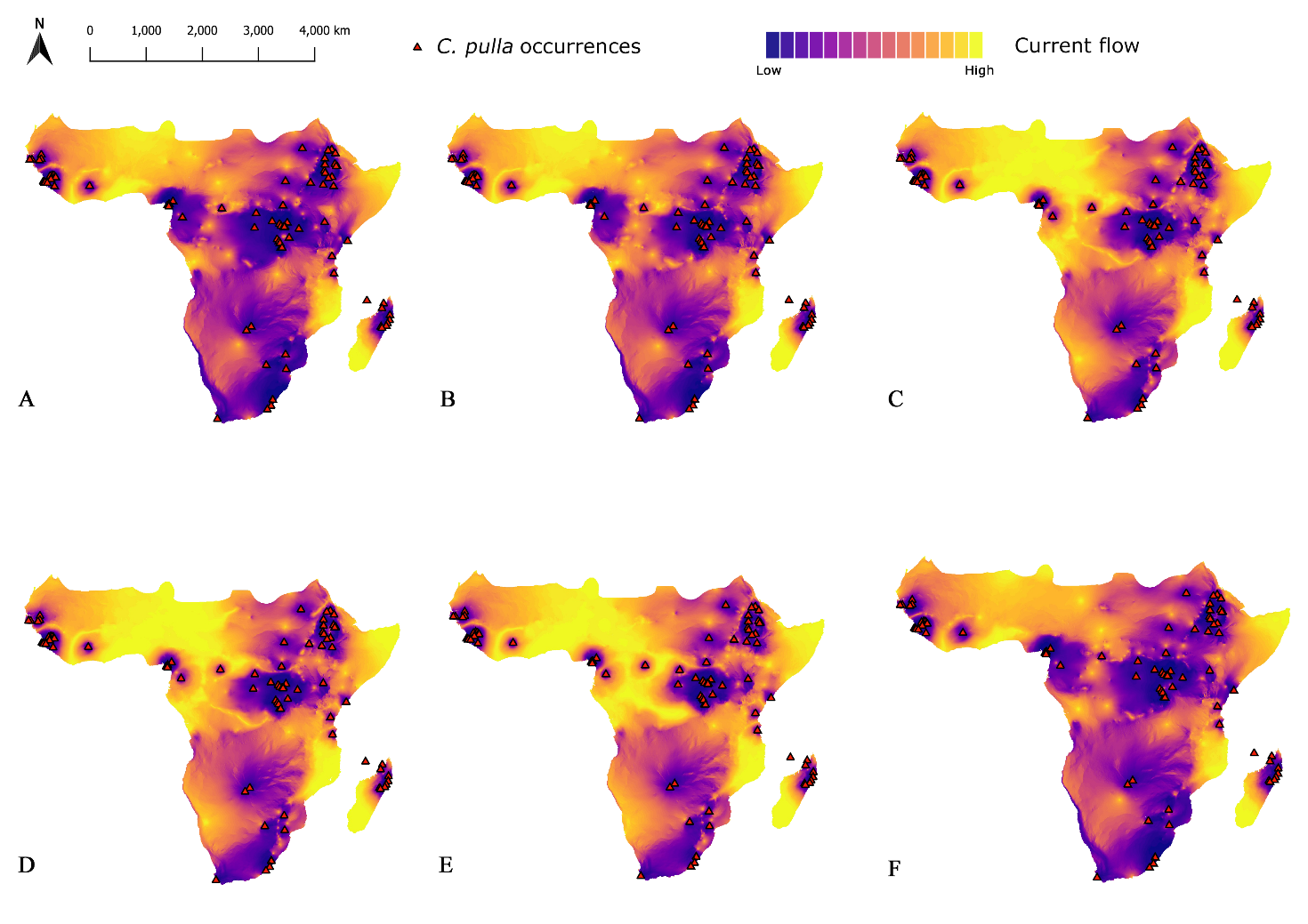


**Fig. S.2 -** Landscape connectivity inferred for future scenarios. (A) SSP2_4.5 2030, (B) SSP2_4.5 2050, (C) SSP2_4.5 2070, (D) SSP3_7.0 2030, (E) SSP3_7.0 2050 and (F) SSP3_7.0 2070. The corridors that allow the *pulla* group to reach the rice cultivations are shown.
